# Synanthropy and ecological aspects of Calliphoridae and Mesembrinellidae (Diptera: Oestroidea) in three ecological areas in Rio de Janeiro State, Brazil

**DOI:** 10.1371/journal.pone.0285844

**Published:** 2023-06-07

**Authors:** Mariana dos Passos Nunes, Wellington Thadeu de Alcantara Azevedo, Alexandre Sousa da Silva, Cláudia Soares dos Santos Lessa, Jeronimo Alencar, Valéria Magalhães Aguiar

**Affiliations:** 1 Departamento de Microbiologia e Parasitologia, Instituto Biomédico, Centro de Ciências Biológicas e da Saúde, Universidade Federal do Estado do Rio de Janeiro, Seropedica, Rio de Janeiro, Brazil; 2 Programa de Pós-Graduação Biologia Animal, Instituto de Biologia e Ciências da Saúde, Universidade Federal Rural do Rio de Janeiro, Seropedica, Brazil; 3 Departamento de Matemática e Estatística, Instituto de Biociências, Centro de Ciências Biológicas e da Saúde, Universidade Federal do Estado do Rio de Janeiro, Seropedica, Brazil; 4 Laboratório de Diptera, Fundação Oswaldo Cruz, Rio de Janeiro, RJ, Brazil; Universiti Teknologi Malaysia - Main Campus Skudai: Universiti Teknologi Malaysia, MALAYSIA

## Abstract

The determination of the synanthropic index is essential to evaluate the degree of association between species, such as diptera and man, based solely on their degree of preference for urban areas. This research aimed to study the synanthropic behavior of Calliphoridae and Mesembrinellidae flies in Rio de Janeiro, Brazil. The experiment was conducted between 2021 and 2022 in three areas, where four traps containing 300 g of fresh liver or with 48 h of putrefaction were installed, remaining exposed for 48 h; after collection the dipterans were sacrificed and taxonomically identified. A total of 2,826 dipterans were collected, represented by nine species of Calliphoridae (89.24%) and ten of Mesembrinellidae (10.76%), with the first record of *Mesembrinella currani* in this biome. The Kruskal-Wallis test showed that the abundance of individuals did not differ among the three analyzed environments. The Mesembrinellidae family was exclusively asynanthrope, along with two species of Calliphoridae: *Hemilucilia benoisti* (Séguy 1925) and *Paralucilia nigrofacialis* (Mello 1969) which were exclusive of the forest area, while Calliphoridae had varied synanthropy. *Lucilia eximia* (Wiedemann 1819) alone represented 57.18% of the total sampled, being the most abundant in all environments except the urban area where *Hemilucilia segmentaria* (Fabricius 1805) totaled 55.73%. No species were exclusive to the urban area, however *Cochliomyia hominivorax* (Coquerel 1858) and *Lucilia cuprina* (Wiedemann 1830) were exclusive to the rural area. The most synanthropic species were *Chrysomya megacephala* (Fabricius 1794) and *Chrysomya albiceps* (Wiedemann 1819).

## Introduction

Synanthropy is the ability that certain species have to take advantage of the anthropic environment, whether obtaining resources such as shelter, water, nutritional or reproductive substrate [1], or colonizing niches unintentionally created by men during the urbanization process, being proportionally dependent on the quantity and quality of resources available there [2]. Several diseases can be transmitted by synanthrope animals (*sin* = around; *anthropos* = man), such as Calliphoridae, which are identified as carriers of enteropathogenic agents. They are able to act as vectors of pathogens such as viruses, bacteria, protozoan cysts and eggs of trematodes, cestodes and nematodes.

Synanthropes can adapt so comfortably to the anthropomorphic environment that they cease to compose significantly relevant populations in their natural habitat [3]. The calculation of the synanthropy indices, therefore, was created to evaluate the association degree between species, such as dipterans and human, based solely on the degree of a species’ preference for the urban area, ranging from + 100 to– 100, where values ranging from +100 to +20 show the association of the studied species with the anthropic environment, and is then classified as synanthrope. Values between +20 and 0 comprise species that show independence from human-inhabited areas, considered hemi-synanthropes; and negative values, between 0 and -100, indicate that this an asynanthrope, by avoiding or distancing itself from urban environments. The formula used is [1]: synanthropy index (S.I.) = (2a + b - 2c) / 2. Where a = percentage of individuals collected in the urban area; b = percentage of individuals collected in the rural area; c = percentage of individuals collected in the forest area. In this formula, the factors depend only on the degree of a species’ preference for areas inhabited by humans, excluding the ability of dipterans to transmit or cause diseases.

The main characteristic of the urban environment is the wide and incessant anthropic activity [4]. Mainly urban and industrial waste, as well as excrement of domestic animals, serve as a substrate for the development of various dipterous species [3]. The rural environment, in turn, is characterized by not having a strong human presence and not being inserted in urban centers, where dipterous species that develop in excrement of domestic animals and animal carcasses predominate [5]. The forest environment has integral domain of the natural landscape. They are usually in protected areas of environmental conservation and do not have a marked presence or anthropic activity, and depending on the level, urbanization can interfere in the resident community [6].

As some of the animals where synanthropy is most expressed belong to the Diptera Order [7], the Calliphoridae family stands out in Rio de Janeiro [5]. Popularly known as blowflies, they are metallic, greenish, bluish, violace and cuprea. The diptera of the Mesembrinellidae family are brownish, and some species may have metallic coloration in the abdomen [8].

Diptera sampling studies are of fundamental importance to analyze ecological behavior, such as population dynamics, diversity, distribution, dispersal areas and seasonality of exotic and native species. As mesembrinelids represent an asynanthrope and bioindicator group [9] and Calliphorids have high adaptability to anthropomorphized environment, with high specimen dispersion and numerous offspring, thus possessing synanthropic species [10], more work on the synanthropy of these two families is needed.

This study aimed to calculate the Synanthropy Index of Calliphoridae and Mesembrinellidae species recorded in three different areas of Atlantic Forest fragments in the State of Rio de Janeiro: (a) forest area–Parque Estadual dos Três Picos, Cachoeiras de Macacu; (b) rural area–cattle ranching on the campus of the Universidade Federal Rural do Rio de Janeiro, Seropédica; and (c) urban area—the campus of the Universidade Federal do Estado do Rio de Janeiro, in Urca; and study the diversity and abundance of Calliphoridae and Mesembrinellidae species, comparing these parameters to the three ecological areas analyzed.

## Materials and methods

**Fig 1:**
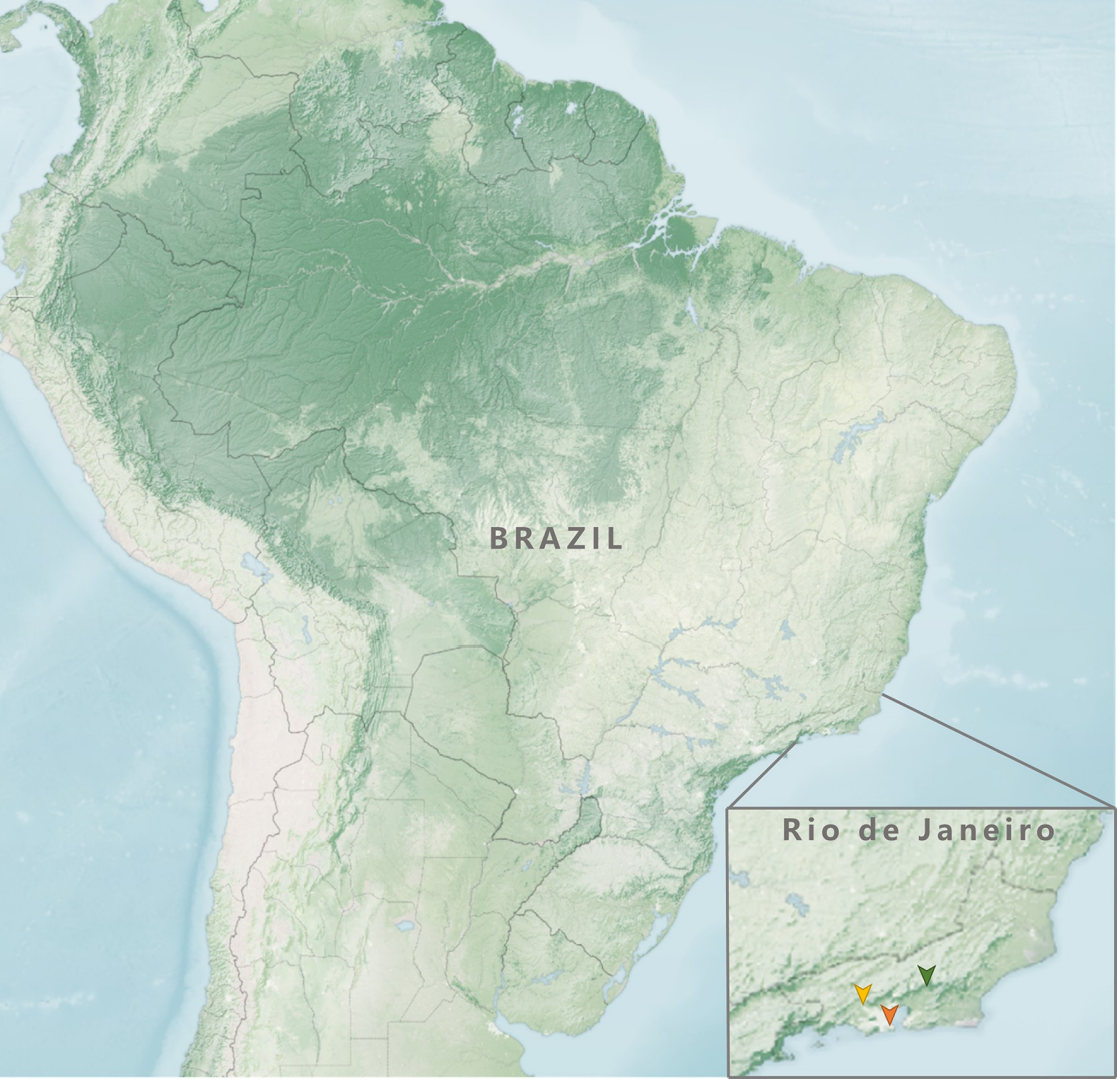
Image of the three ecological areas of the Rio de Janeiro State where the research was conducted: Forest area (demarcated in green) at the Parque Estadual dos Três Picos; rural area (demarcated in yellow) cattle ranchng on the campus of the Universidade Federal Rural do Rio de Janeiro; and urban area (demarcated in red) campus of the Universidade Federal do Estado do Rio de Janeiro, Urca district.

The collections were carried out in four geo-referenced points (Fig 1, Table 1) of the three ecological areas evaluated in Rio de Janeiro. A trap was installed at each point to capture dipterans. The forest area chosen for this research was the Parque Estadual dos Três Picos (PETP), a conservation unit located in Serra do Mar, a mountainous region of Rio de Janeiro. Authorization for the scientific research in this conservation unit was given from the Instituno Estadual do Ambiente (INEA), n° 019/2020. The rural area is located in the Universidade Federal Rural do Rio de Janeiro (UFRRJ) in Seropédica. The urban area chosen for this study was the Universidade Federal do Estado do Rio de Janeiro (UNIRIO), campus located in the Urca district, Rio de Janeiro.

**Table 1:** Geo-referenced sites and altitude of the ecological areas studied in the Rio de Janeiro State. Forest area—Parque Estadual dos Três Picos; rural area–cattle ranching on the campus of the Universidade Federal Rural do Rio de Janeiro; and urban area—campus of the Universidade Federal do Estado do Rio de Janeiro, Urca district.

Sites	Florestal	Rural	Urban
**Site 1**	22°25’07,13"S 42°36’11,97"O	22°45’40,42"S 43°42’09,23"O	22°57’19,42"S 43°10’08,56"O
	484 m	28 m	17 m
**Site 2**	22°25`07,75"S 42°36`11,09"O	22°45’41,01"S 43°42’09,84"O	22°57’18,93"S 43°10’10,17"O
	496 m	28 m	19 m
**Site 3**	22°25`07,06"S 42°36`09,92"O	22°45’40,57"S 43°42’09,67"O	22°57’16,86"S 43°10’08,99"O
	519 m	28 m	11 m
**Site 4**	22°25’06,17"S 42°36`09,80"O	22°45’39,83"S 43°42’09,57"O	22°57’17,30"S 43°10’10,99"O
	532 m	28 m	17 m

**Fig 2:**
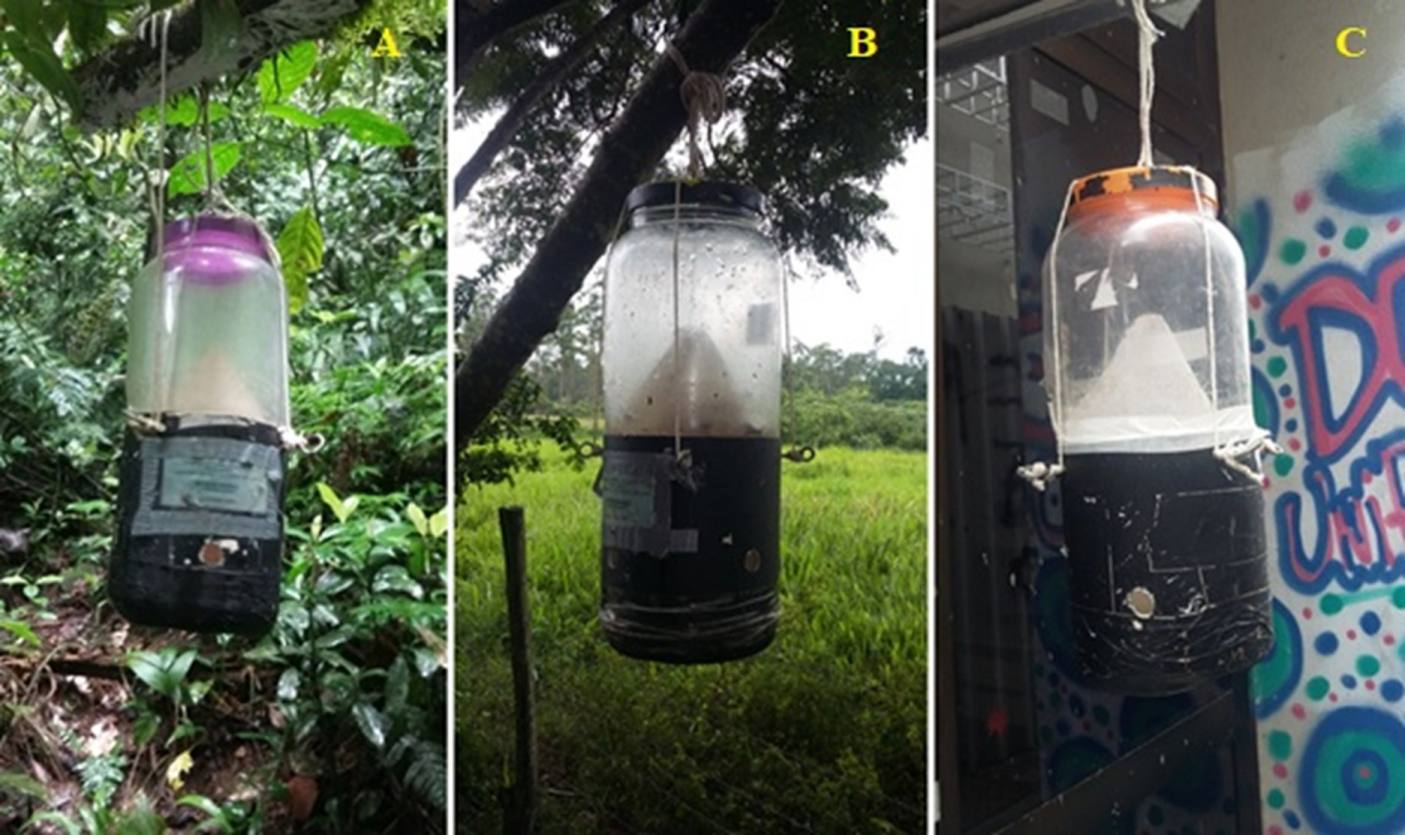
Ilustration of the traps described by [11] in A: forest area (Parque Estadual dos Três Picos—Cachoeiras de Macacu), in B: rural area (Universidade Federal Rural do Rio de Janeiro—Seropédica) and in C: urban area (Universidade Federal do Estado do Rio de Janeiro, Urca campus, Rio de Janeiro).

The experiment was carried out between June 2021 and February 2022. In each ecological area, four traps made of PVC were installed (Fig 2), following the model described by [11]. The attractive bait was placed at the base of the traps. Bovine liver in two stages of decomposition was used as bait: two traps containing 300 grams of fresh bait and two traps containing 300 grams of bait with 48 hours of putrefaction. The collected dipterans were sacrificed in solution based on ethyl alcohol and ethyl acetate. Then, they were sent to the Laboratório de Estudos de Dípteros (LED-UNIRIO) where they were transferred with their respective identification to petri dishes lined with absorbent paper, sealed with PVC film and stored in a freezer at 4°C until sorting.

The insects were then identified under incident light using stereoscopic microscopes (Olympus SZX7), following taxonomic keys [12, 13] and with the collaboration of Prof. Dr. Rubens Pinto de Mello, Fundação Oswaldo Cruz, and then pinned and stored in the entomological collections of the Museu Nacional and LED-UNIRIO.

The Synanthropy Index was calculated using the formula proposed by [1]: **S.I.** = (**2a + b – 2c**)/**2**, where **a** = percentage of individuals collected in the urban area; **b** = percentage of individuals collected in the rural area; **c** = percentage of individuals collected in the forest area, employing the same collection method for all areas and evaluating each species, in order to analyze the degree of association between the dipteran species and the anthropic environment. The Shapiro-Wilk normality test was used to evaluate the abundance variable, and as there was no normality in the data, the use of nonparametric tests was adopted. Kruskal-Wallis and Wilcoxon tests, which compare independent samples, were used to assess the influence of abundance in the three environments, evaluating the degree of association between the variables, considering a 5% significance level for the tests [14, 15]. Data is available from Open Science Framework.

## Results

**Fig 3:**
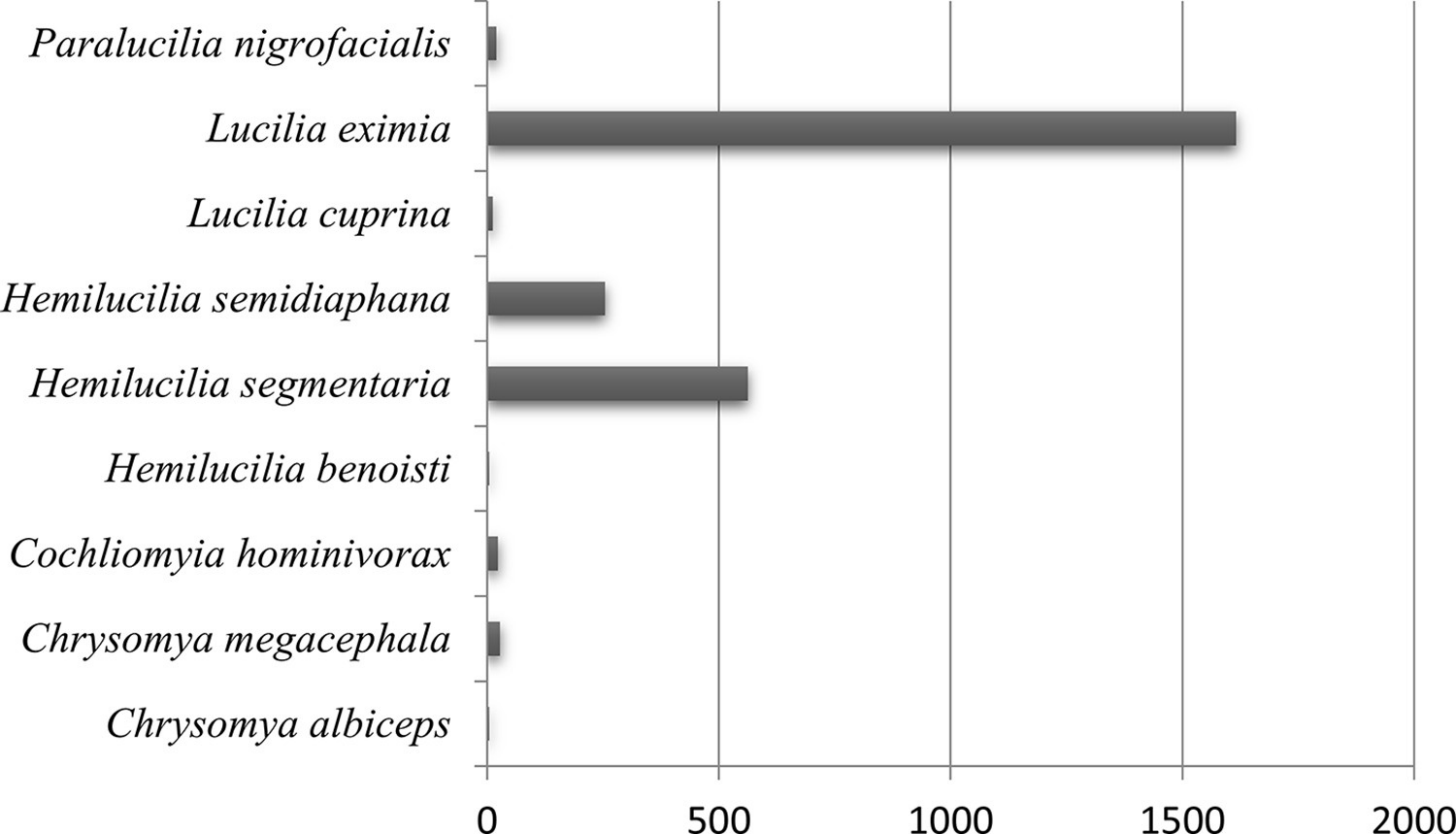
Abundance of Calliphoridae species collected in the forest area (Parque Estadual dos Três Picos–Cachoeiras de Macacu), rural area (Universidade Federal Rural do Rio de Janeiro—Seropédica) and urban area (Universidade Federal do Estado do Rio de Janeiro, Urca), in the four seasons between 2021 and 2022.

**Fig 4:**
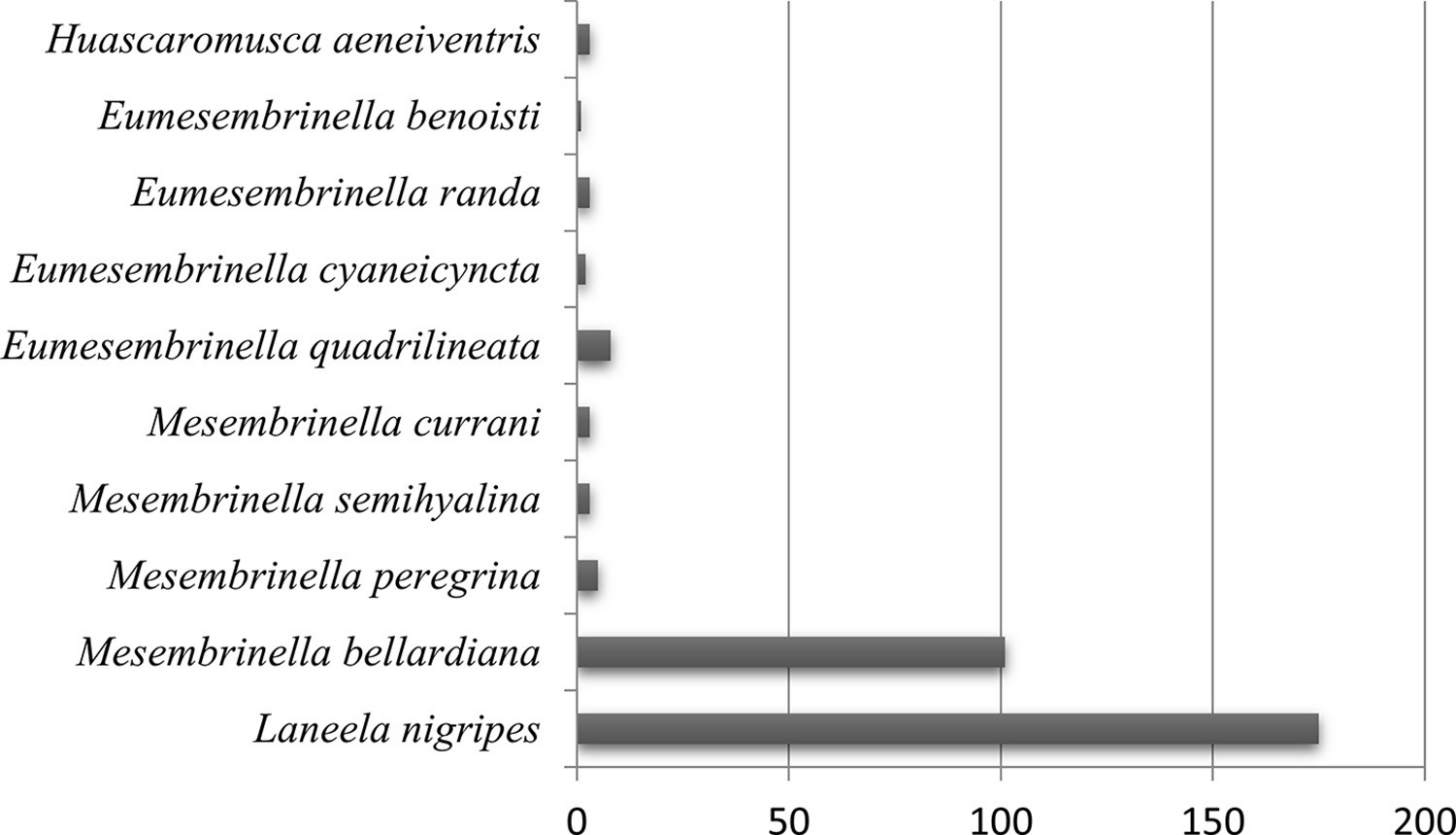
Abundance of Mesembrinellidae species collected in the forest area (Parque Estadual dos Três Picos–Cachoeiras de Macacu), rural area (Universidade Federal Rural do Rio de Janeiro—Seropédica) and urban area (Universidade Federal do Estado do Rio de Janeiro, Urca), in the four seasons between 2021 and 2022.

**Fig 5:**
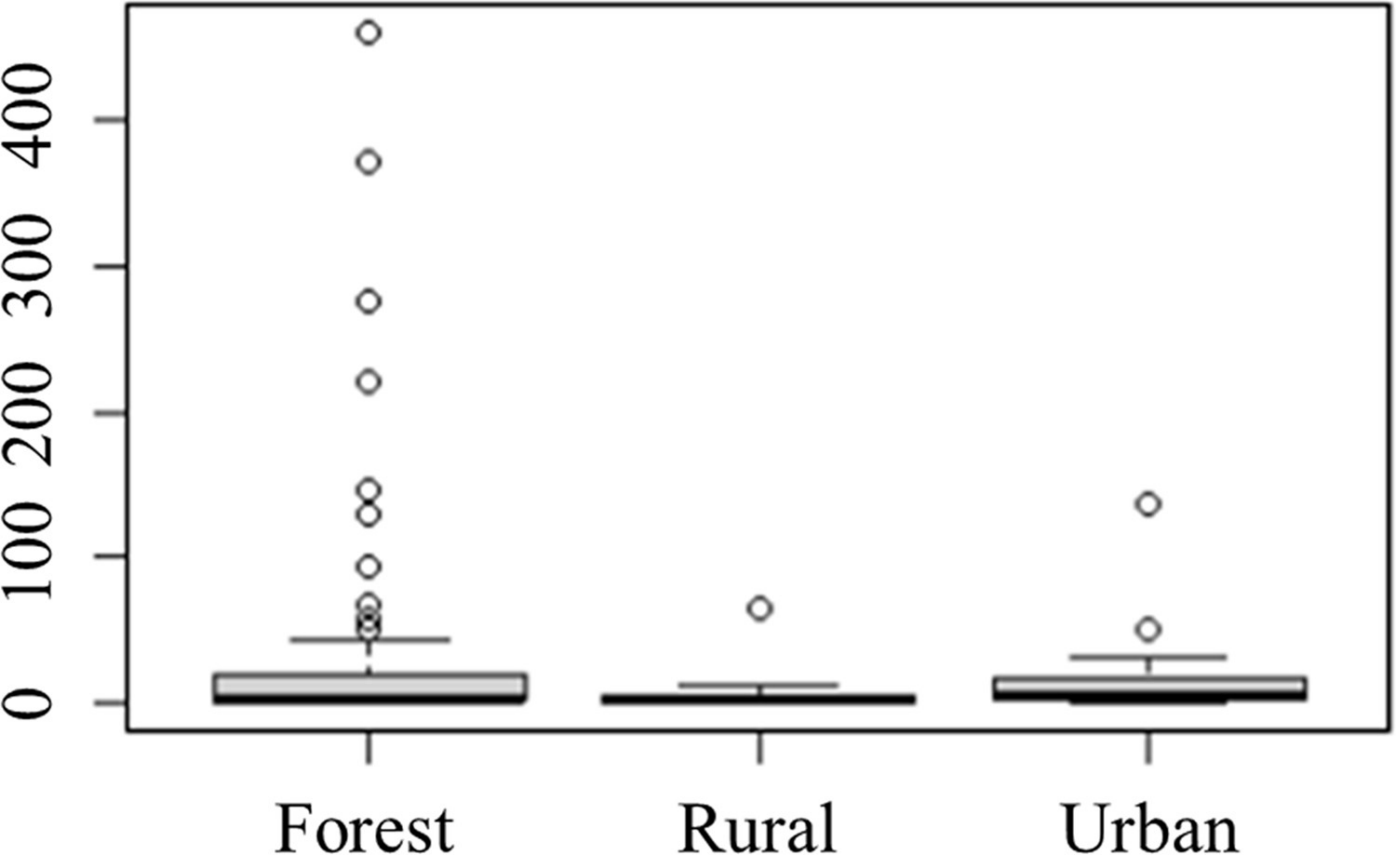
*Box Plot* of the abundance of Calliphoridae and Mesembrinellidae species collected in the forest area (Parque Estadual dos Três Picos–Cachoeiras de Macacu), rural area (Universidade Federal Rural do Rio de Janeiro—Seropédica) and urban area (Universidade Federal do Estado do Rio de Janeiro, Urca), in the four seasons between 2021 and 2022.

A total of 2,826 dipterans were collected from May 2021 to February 2022 and were represented by 19 species of the Calliphoridae (89.24%) and Mesembrinellidae (10.76%) families. As shown in Table 2, Figs 3 and 4, the forest environment was the most representative where the main Calliphoridae species were *Lucilia eximia* Wiedemann, 1819 (61.95%) and *Hemilucila segmentaria* (14.81%), while the main Mesembrinellidae species were *Mesembrinella bellardiana* (7.60%) and *Mesembrinella peregrina* (4.39%) and *Mesembrinella currani* (0,10%), the latest with its first record in the Atlantic Forest. In the urban environment, the two most representative species were *Hemilucilia segmentaria* (55.73%) and *Lucilia eximia* (26.56%). In the rural area, the two most representative species were *Lucilia eximia* (62.86%) and *Cochliomyia hominivorax* (16.43%). The Kruskal-Wallis test was performed to analyze whether the species’ abundance varied between the environments, showing no statistical difference (chi- square = 3.7854; p-value = 0.1507) (Fig 5).

**Table 2:** Absolute abundance (n) and relative frequency (%) of Calliphoridae and Mesembrinellidae in the three environments: Forest area (Parque Estadual dos Três Picos–Cachoeiras de Macacu), rural area (Universidade Federal Rural do Rio de Janeiro—Seropédica) and urban area (Universidade Federal do Estado do Rio de Janeiro, Urca), in the four seasons between 2021 and 2022.

Species	Forest		Rural		Urban		Total	
CALLIPHORIDAE	**n**	**%**	**n**	**%**	**n**	**%**	**n**	**%**
*Chrysomya albiceps* Wiedemann, 1819	-	-	3	2.14	1	0.26	4	0.14
*Chrysomya megacephala* Fabricius, 1794	-	-	7	5	20	5.21	27	0.95
*Cochliomyia hominivorax* Coquerel, 1858	-	-	23	16.43	-	-	23	0.81
*Hemilucilia benoisti* Séguy, 1925	4	0.17	-	-	-	-	4	0.14
*Hemilucilia segmentaria* Fabricius, 1805	341	14.81	7	5	214	55.73	562	19.89
*Hemilucilia semidiaphana* Rondani, 1850	207	8.99	-	-	47	12.24	254	8.99
*Lucilia cuprina* Wiedemann, 1830	-	-	12	8.57	-	-	12	0.42
*Lucilia eximia* Wiedemann, 1819	1426	61.95	88	62.86	102	26.56	1616	57.18
*Paralucilia nigrofacialis* Mello, 1969	20	0.86	-	-	-	-	20	0.70
MESEMBRINELLIDAE								
*Laneela nigripes* Guimarães, 1977	175	7.60	-	-	-	-	20	6.19
*Mesembrinella bellardiana* Aldrich, 1922	101	4.39	-	-	-	-	175	3.57
*Mesembrinella peregrina* Aldrich, 1922	5	0.21	-	-	-	-	101	0.17
*Mesembrinella semihyalina* Mello, 1967	3	0.13	-	-	-	-	5	0.10
*Mesembrinella currani* Guimarães, 1977	3	0.13	-	-	-	-	3	0.10
*Eumesembrinella quadrilineata* Fabricius, 1805	8	0.34	-	-	-	-	3	0.28
*Eumesembrinella cyaneicyncta* Surcouf, 1919	2	0.08	-	-	-	-	8	0.07
*Eumesembrinella randa* Walker, 1849	3	0.13	-	-	-	-	2	0.10
*Eumesembrinella benoisti* Séguy, 1925	1	0.04	-	-	-	-	1	0.03
*Huascaromusca aeneiventris* Wiedemann, 1830	3	0.13	-	-	-	-	3	0.10
**Total**	**2302**	**100**	**140**	**100**	**384**	**100**	**2826**	**100**

**Fig 6:**
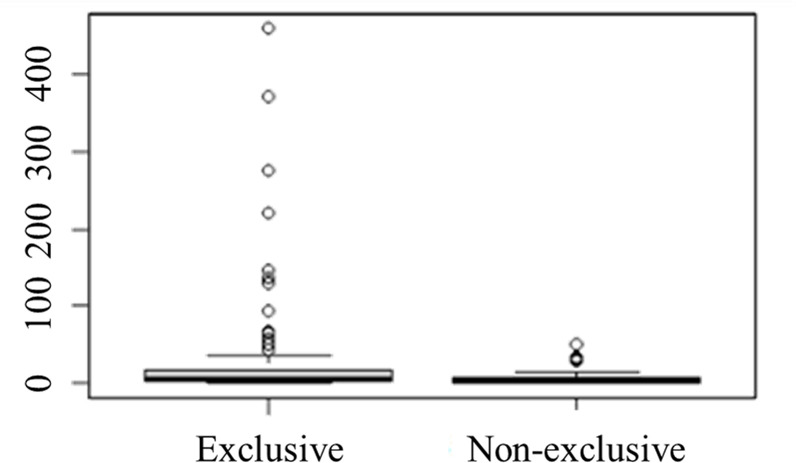
*Box Plot* comparing the abundance of Calliphoridae and Mesembrinellidae species that were exclusive and non-exclusive to the forest area (Parque Estadual dos Três Picos–Cachoeiras de Macacu in the four seasons between 2021 and 2022. Aversion to inhabited areas.

Only *Cochliomyia hominivorax* and *Lucilia cuprina* were exclusive to rural areas, while all Mesembrinellidae species and two Calliphoridae species (*Paralucilia nigrofacialis* and *Hemilucilia benoisti*) were exclusive to forest area (Table 3). No species was exclusive to the urban area. The Wilcoxon test was performed to compare the abundance of individuals collected in forest areas comparing those which occurred exclusively in this environment and those whose occurrence was not exclusive to this environment, showing that the abundance of exclusive species to this environment happens to be greater than those of species that are not exclusive (W = 2400.5, p-value = 0.05294) (Fig 6).

**Table 3:** Exclusivity of Calliphoridae and Mesembrinellidae species in the forest area (Parque Estadual dos Três Picos–Cachoeiras de Macacu) (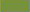), rural area (Universidade Federal Rural do Rio de Janeiro—Seropédica) (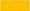) and urban area (Universidade Federal do Estado do Rio de Janeiro, Urca) (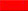), in the four seasons between 2021 and 2022.

Species		Exclusive from		
		Forest	Rural	Urban
CALLIPHORIDAE	*Chrysomya albiceps*			
	*Chrysomya megacephala*			
	*Cochliomyia hominivorax*			
	*Hemilucilia benoisti*			
	*Hemilucilia segmentaria*			
	*Hemilucilia semidiaphana*			
	*Lucilia cuprina*			
	*Lucilia eximia*			
	*Paralucilianigrofacialis*			
MESEMBRINELLIDAE	*Laneela nigripes*			
	*Mesembrinella bellardiana*			
	*Mesembrinella peregrina*			
	*Mesembrinella semihyalina*			
	*Mesembrinella currani*			
	*Eumesembrinella quadrilineata*			
	*Eumesembrinella cyaneicyncta*			
	*Eumesembrinella randa*			
	*Eumesembrinella benoisti*			
	*Huascaromusca aeneiventris*			
**Total**		**12**	**2**	**0**

**Fig 7:**
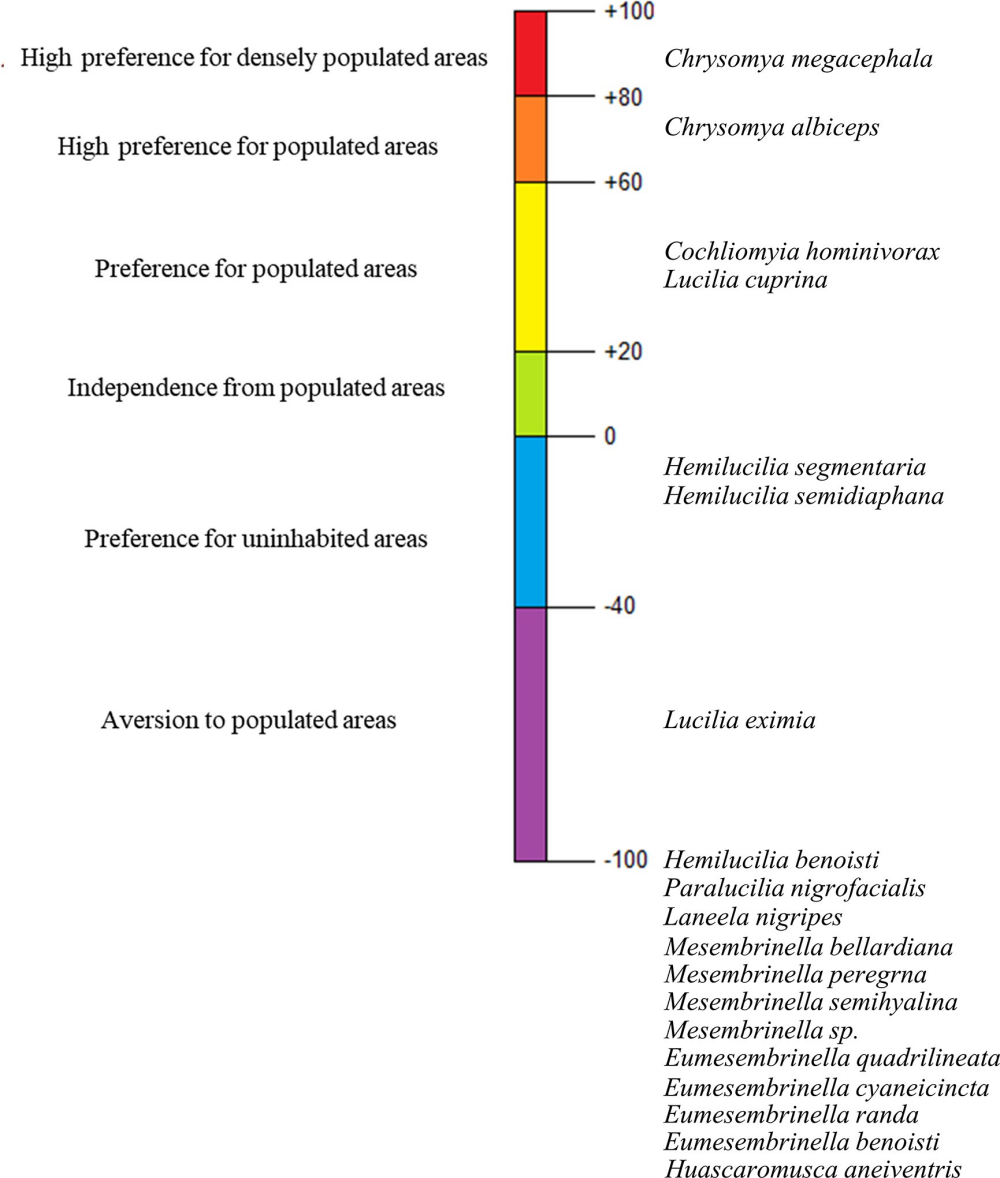
Average synanthropy of all Calliphoridae and Mesembrinellidae species collected in the forest area (Parque Estadual dos Três Picos–Cachoeiras de Macacu), rural area (Universidade Federal Rural do Rio de Janeiro—Seropédica) and urban area (Universidade Federal do Estado do Rio de Janeiro, Urca), in the four seasons between 2021 and 2022.

Notably, when analyzing the synanthropy of all Calliphoridae and Mesembrinellidae species collected throughout the four seasons (autumn 2021, winter 2021, spring 2021 and summer 2022), all representative species of the Mesembrinellidae family were fully asynanthropes (Fig 7), along with two Calliphoridae species: *H*. *benoisti* and *P*. *nigrofacialis*. The most synanthrope species were *C*. *megacephala* and *C*. *albiceps*, followed by *Co*. *hominivorax* and *L*. *cuprina*. When reaching more negative spectra we found *H*. *segmentaria* and *H*. *semidiaphana*, while *L*. *eximia* was somewhat more asynanthrope than the last two mentioned species.

## Discussion

As the dipterans of the Calliphoridae and Mesembrinellidae families are virtually present in both natural, rural and urban areas [16] it is of fundamental importance to study their population composition in a given biome. Through this acquired knowledge of the abundance, richness and endemicity, it is possible to develop subsidies that generate measures to preserve environments, indicating endemic species, and those that may cause myiasis or transmit several pathogens, and that can be used in environmental entomology in the Atlantic Forest.

The richness and abundance of Calliphoridae species are favored in proportion to the agglomeration of garbage and organic matter in anthropic environments [1], while the opposite is observed for Mesembrinellidae. Comparing the forest environment with the urban and rural ones, the first environment showed higher richness and abundance indexes than the other two, ranging from eight to ten species in the forest environment, against two to five species in the urban and rural environments. Each environment allows different niches and ecological interactions between organisms that are given by the characteristics of each place, and it is these differences that generate variability in the distribution of species [17]. Therefore, there are divergences in the data regarding species diversity in a given environment, where [18] showed that diversity is greater in the anthropic environment, while [19] revealed it is higher in the preserved environment.

The species of the Calliphoridae family have varied synanthropy, with species present at all different levels. As in the genus *Lucilia*, which in the present study the species *L*. *eximia* presented negative synanthropy, while *L*. *cuprina* presented positive synanthropy being eusynanthropes. In the first case it differs from that found by [20], while in the second case it corroborates with this author. The genus *Chrysomya* was the most synanthrope in this study, agreeing with [21]. However, for *C*. *albiceps*, the study of [22] showed it to be rare in urban environments.

*H*. *benoisti* and *P*. *nigrofacialis* were the only Calliphoridae species found exclusively in the forest environment. The genus *Paralucilia* is common in preserved environments [23, 24]. In this study, *P*. *nigrofacialis* showed the lowest level of synanthropy, being exclusive to the forest environment, according to the literature cited above. The same goes for *Hemilucilia benoisti*, found mainly in forest areas [5]. However, some authors such as [25] stated that the species *H*. *semidiaphana* is independent of urban areas, while most authors agree that *H*. *segmentaria* prefers forest environments, although it is also present in rural and urban areas, presenting a low synanthropy index [10]. In this study, both *H*. *semidiaphana* and *H*. *segmentaria* presented negative synanthropy, but these were the two species whose index varied most within the seasons.

*Cochliomyia hominivorax* was considered synanthrope, being exclusive to the rural area, as well as *L*. *cuprina*. According to [26], *Co*. *hominivorax* is the main cause of primary myiasis in the city of Rio de Janeiro, but in this study the species in question was not collected in the urban area.

*Laneela nigripes* and *Mesembrinella bellardiana* were considered the most abundant in less anthropic areas. These data corroborate with [24, 27, 28]. Several studies over the decades portray the Mesembrinellidae family as asynanthrope, being found exclusively in forest areas or deeper spots in forest fragments [29]. The present study corroborates this hypothesis, as all species of this family showed the lowest degree of synanthropy, including *M*. *currani*, previously recorded in the State of Pará, which belongs to the Amazon Forest biome [30]. In our study this species was recorded for the first time in the State of Rio de Janeiro, which belongs to the Atlantic Forest biome, occurring strictly in preserved forest area and low abundance.

Due to the growing and disorderly expansion of urban areas that cause the emergence of new niches and habitats, it is essential to know the dipterans that occur in the Atlantic Forest of Rio de Janeiro to develop strategies and measures that protect this fauna and, consequently, its habitat [31]. Thus, we conclude that all species of the Mesembrinellidae family are exclusively asynanthropes, while the Calliphoridae species show varied synanthropy, with species present at all different levels of synanthropy. Regarding the diversity of Calliphoridae and Mesembrinellidae species, we conclude that these dipterans occurred in the three environments totaling nine different species for Calliphoridae and ten for Mesembrinellidae, with the first family accounting for almost 90% of the total sampled, with *L*. *eximia* accounting for more than 57% of the total. Subsequent studies are expected to analyze ecological areas comparatively distinct from each other, helping to understand more broadly the knowledge of the Calliphoridae and Mesembrinellidae families.
